# A Case Report of a Cold Chest Wall Abscess Following Bacillus Calmette-Guérin Vaccination

**DOI:** 10.7759/cureus.51942

**Published:** 2024-01-09

**Authors:** Syed Rizwan Hussain, Sarah Rayyani, Dania Fatani, Abdulrhman Almas, Ahlam Alharbi

**Affiliations:** 1 Internal Medicine, Al-Ameen Hospital, Taif, SAU; 2 General Practice, Jazan University, Jazan, SAU; 3 General Practice, Arabian Gulf University, Manama, BHR; 4 General Practice, King Faisal University, Hofuf, SAU; 5 Family Medicine, Primary Health Care Center, Riyadh, SAU

**Keywords:** anti-tuberculosis therapy, computed tomography, mycobacterium tuberculosis, bcg vaccine, chest wall abscess

## Abstract

The Bacillus Calmette-Guérin (BCG) vaccine, a cornerstone in global immunization programs for tuberculosis prevention, has generally proven to be safe and effective. However, rare complications, including localized abscess formation, have been reported. This case report highlights a two-year-old male who developed a painless swelling on the left chest wall, noticed six weeks post-BCG vaccination. Physical and imaging evaluations confirmed a cold abscess. Laboratory tests ruled out alternative diagnoses. Antitubercular therapy led to a favorable response, avoiding surgical intervention. Follow-up revealed complete resolution, showcasing successful management of this rare BCG-related complication in a pediatric patient. The success of antimycobacterial therapy supports a tailored and conservative approach, raising questions about the necessity of surgical intervention. The presented case sheds light on the complex interplay between BCG vaccination, host response, and rare complications, providing valuable insights for further research. Vigilance, robust surveillance, and collaborative efforts are essential to unravel vaccine-related adverse events. This case contributes to a deeper understanding of rare BCG-related complications, guiding clinical practice, and advancing the knowledge base.

## Introduction

The Bacillus Calmette-Guérin (BCG) vaccine, introduced nearly a century ago for the prevention of tuberculosis, has been a cornerstone of global immunization programs. Despite its overall safety and efficacy, rare complications, such as localized abscess formation, have been documented [1]. BCG-related complications are typically localized and self-limiting, ranging from regional lymphadenitis to more uncommon manifestations like abscess formation [1,2]. The occurrence of a cold abscess at a site distant from the vaccination site, as observed in this case, adds a distinctive dimension to the spectrum of BCG-related complications [1]. Understanding the pathophysiological mechanisms underlying such occurrences and delineating the optimal management strategies is crucial for clinicians faced with this uncommon presentation.

This case presents a unique scenario of a two-year-old male child who developed a cold abscess on the left chest wall following BCG vaccination, an unusual and infrequently reported complication. By presenting a detailed account of the clinical course, diagnostic challenges, and successful therapeutic interventions, this report contributes to the expanding body of knowledge surrounding BCG-related complications in pediatric patients.

## Case presentation

A two-year-old male child presented to the pediatric outpatient department with a chief complaint of a progressively enlarging, painless swelling on the left chest wall. The parents reported noticing the swelling approximately six weeks ago, and it had gradually increased in size. The child had a history of receiving BCG vaccination at birth as part of the routine immunization schedule. No other significant medical or surgical history was reported, and the child had been meeting developmental milestones appropriately. The family denied any history of trauma or recent infections, and there was no family history of immunodeficiency or tuberculosis.

Upon physical examination, a non-tender, soft fluctuant swelling was palpated on the left chest wall. The overlying skin was erythematous and warm and demonstrated signs of inflammation. The child was afebrile, with no respiratory distress or other systemic symptoms. The regional lymph nodes were non-palpable, and the remainder of the physical examination was unremarkable. Laboratory investigations, including complete blood count and inflammatory markers, were within normal limits (Table 1).

**Table 1:** Initial laboratory investigations with reference range

Laboratory Parameters	Results	Reference Range
Hemoglobin	12.5 g/dL	11.5 - 15.5 g/dL
White Blood Cell Count	8,000 cells/mm³	4,000 - 11,000 cells/mm³
Platelet Count	250 x 10³/µL	150 - 450 x 10³/µL
Blood Glucose	90 mg/dL	70 - 100 mg/dL
Blood Urea Nitrogen	15 mg/dL	7 - 20 mg/dL
Creatinine	0.8 mg/dL	0.6 - 1.2 mg/dL
Sodium	138 mmol/L	135 - 145 mmol/L
Potassium	4.2 mmol/L	3.5 - 5.1 mmol/L
Aspartate Aminotransferase	30 U/L	10 - 40 U/L
Alanine Aminotransferase	25 U/L	7 - 56 U/L
Total Bilirubin	0.6 mg/dL	0.3 - 1.2 mg/dL
Alkaline Phosphatase	80 U/L	40 - 130 U/L
Blood Urea Nitrogen	18 mg/dL	7 - 20 mg/dL
Creatinine	0.9 mg/dL	0.6 - 1.2 mg/dL
Erythrocyte Sedimentation Rate	10 mm/hour	0 - 20 mm/hour
C-Reactive Protein	5 mg/L	0 - 5 mg/L
Prothrombin Time	12 seconds	10 - 14 seconds
International Normalized Ratio	1.1	0.9 - 1.1

The differential diagnosis at this point included infectious etiologies such as Staphylococcal or Streptococcal abscess. Immunodeficiency disorders were also considered given the site and nature of the lesion. A detailed history ruled out any signs of primary immunodeficiency or recurrent infections in the child.

**Figure 1:**
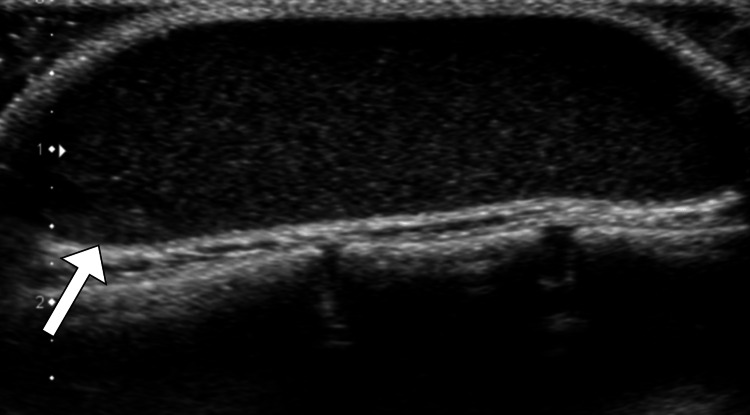
Ultrasound image illustrating the left chest wall lesion The hypoechoic, fluid-filled cavity with internal echoes is evident, indicative of the cold abscess.

**Figure 2:**
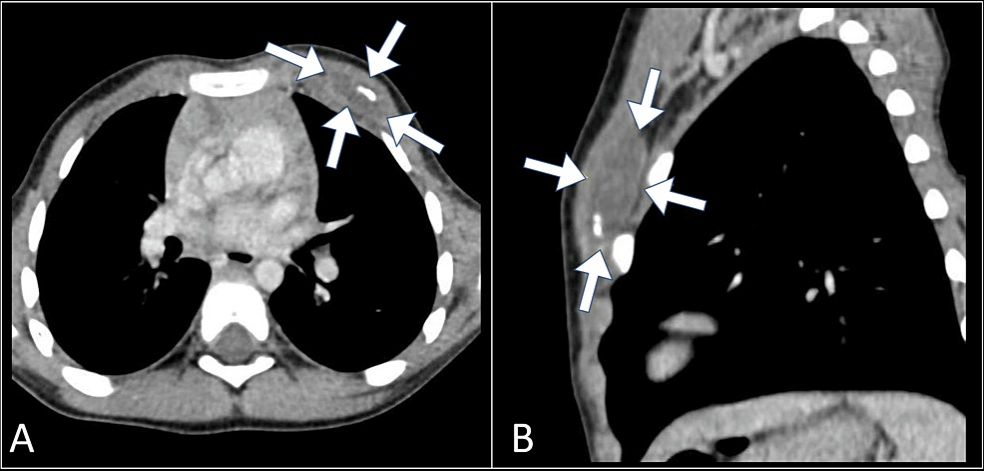
Axial (A) and sagittal (B) CT images revealing a collection (arrows) within the left chest wall CT: computed tomography

Ultrasound examination demonstrated a well-defined, hypoechoic collection within the left chest wall (Figure 1). This result prompted further imaging studies, leading to a contrast-enhanced computed tomography scan. The computed tomography scan revealed a radiolucent lesion involving the subcutaneous tissue and underlying pectoralis muscles on the left anterior chest wall, providing a more detailed understanding of the abscess (Figure 2).

Given the clinical suspicion of mycobacterial infection, an ultrasound-guided aspiration of the lesion was performed. The aspirate revealed purulent material and subsequent acid-fast staining and culture identified Mycobacterium, confirming the diagnosis of a cold abscess secondary to BCG vaccination.

The child was promptly started on antitubercular therapy consisting of isoniazid, rifampicin, and pyrazinamide. Surgical intervention was deferred, and the patient was closely monitored for clinical response and adverse effects. Over the hospital course, the child demonstrated a favorable response to therapy, with a reduction in the size of the abscess and resolution of inflammatory signs.

Follow-up evaluations included clinical assessment, repeat imaging studies, and monitoring for potential adverse effects of antitubercular therapy. The child continued to thrive, with complete resolution of the abscess over subsequent months.

## Discussion

The observed cold abscess in this case, although a rare sequel of BCG vaccination, underscores the need for vigilance in monitoring adverse events associated with immunization. The pathophysiological mechanisms leading to this manifestation remain elusive, warranting further investigation. Potential hypotheses may involve an aberrant immune response to the mycobacterial components of the vaccine, triggering a delayed-type hypersensitivity reaction that manifests as a distant cold abscess [1]. This unique presentation prompts consideration of individual host factors, immunogenetic predispositions, or variations in vaccine strain virulence.

Diagnostic challenges, in this case, were accentuated by the unexpected normalcy of the chest X-ray, necessitating a more advanced imaging modality, such as a contrast-enhanced CT scan, for accurate characterization. This emphasizes the importance of a stepwise diagnostic approach and the judicious use of imaging modalities to avoid delays in diagnosis.

The successful management of this case highlights the efficacy of antimycobacterial therapy, obviating the need for surgical intervention [2,3]. However, the optimal duration of therapy and potential long-term sequelae, especially in pediatric populations, remain areas of ongoing research. The decision to defer surgical intervention in this case was supported by the clinical response to pharmacotherapy [2-4], affirming the importance of a tailored and conservative approach in selected cases.

Moreover, this case underscores the necessity for continuous monitoring of vaccine safety, especially given the global efforts to expand immunization programs [3,5]. The rarity of such complications necessitates collaboration among healthcare providers, researchers, and vaccine manufacturers to gather and analyze data systematically. This case report adds valuable clinical data to the existing literature, contributing to a more comprehensive understanding of rare BCG-related complications in the pediatric population.

## Conclusions

In conclusion, the intricate interplay between immunization, host response, and rare complications presented in this case offers novel insights and prompts further investigation into the underlying mechanisms. Continued vigilance, robust surveillance systems, and collaborative research efforts are pivotal for unraveling the complexities of vaccine-related adverse events. The comprehensive analysis of this case contributes to the existing body of knowledge, fostering a deeper understanding of rare BCG-related complications and guiding clinical practice.

## References

[1] Aribas OK, Kanat F, Gormus N, Turk E (2002). Cold abscess of the chest wall as an unusual complication of BCG vaccination. Eur J Cardiothorac Surg.

[2] Kim DH, Choi CW (2009). Chest wall abscess likely due to BCG vaccination in a child. Infection.

[3] Lee HS, Seo KJ, Kim JJ (2015). Chest wall granuloma associated with BCG vaccination presenting as hot abscess in an immunocompetent infant. J Cardiothorac Surg.

[4] Olgun KA, Fikret K, Niyazi G, Turk E (2002). Cold abscess of the chest wall as an unusual complication of BCG vaccination. Eur J Cardiothorac Surg.

[5] Sedighi P, Sadrosadat ST, Movahedi M, Sedighi I (2022). BCG-Induced cold abscess as a complication of inadvertent vaccine injection: a case series. Clin Case Rep.

